# Application of Tinel’s test combed with clinical neurosensory test distinguishes spontaneous healing of lingual nerve neuropathy after mandibular third molar extraction

**DOI:** 10.1186/s40902-023-00389-3

**Published:** 2023-06-19

**Authors:** Shigeyuki Fujita, Itaru Tojyo, Shigeru Suzuki, Fumihiro Tajima

**Affiliations:** 1 Oral and Maxillofacial Surgery, Wakayama Medical University, Kimiidera 811-1, Wakayama City, 641-8509 Japan; 2 Rehabilitation Medicine, Wakayama Medical University, Kimiidera 811-1, Wakayama City, 641-8510 Japan

**Keywords:** Mandibular third molar extraction, Lingual nerve disturbance, Spontaneous healing, Tinel’s test, Clinical neurosensory testing, Microneurosurgery

## Abstract

**Background:**

Extraction of the mandibular third molar, the most frequent and important surgical procedure in the clinical practice of oral surgery, is associated with the risk of injury of the lingual nerve. Neuropathy of the lingual nerve poses diagnostic challenges regarding the transient or permanent nature of the injury. No consensus or criteria have been established regarding the diagnosis of lingual nerve neuropathy. We applied both Tinel’s test and clinical neurosensory testing together, which can be easily used at the bedside in the early stages of injury. Therefore, we propose a new method to differentiate between lesions with the ability to heal spontaneously and those that cannot heal without surgery.

**Results:**

Thirty-three patients (29 women,  4 men; mean age, 35.5 years) were included in this study. For all patients, the median interval between nerve injury and initial examination was 1.6 months and that between nerve injury and the second examination before determining the need for surgical management was 4.5 months. The patients were assigned to either group A or B. The spontaneous healing group (group A, *n* = 10) revealed a tendency for recovery within 6 months after tooth extraction. In this group, although there were individual differences in the degree of recovery, a remarkable tendency for recovery was observed based on clinical neurosensory testing in all cases. None of the patients were diagnosed with allodynia. In seven cases, the Tinel test result was negative at the first inspection, and in three cases, the result changed to negative at the second inspection. Conversely, in group B(*n* = 23), no recovery trend was observed with regard to clinical neurosensory testing, and nine patients had allodynia. Further, the Tinel test result was positive for all patients in both examinations.

**Conclusions:**

Our findings indicate that in case of transient lingual nerve paralysis, clinical neurosensory testing findings deteriorate immediately after tooth extraction and gradually recover, while Tinel’s test shows a negative result. Using Tinel’s test and clinical neurosensory testing together enabled early and easy identification of the severity of the lingual nerve disorder and of lesions that would heal spontaneously without surgical management.

## Background

Lingual nerve  (LN) disturbance is a rare peripheral neuropathy occurring after extraction of a mandibular third molar [1–4]. Lingual neuropathy is more difficult to treat than inferior alveolar neuropathy [5, 6]. LN disturbance has several symptoms, including analgesia, with no pain even on accidentally biting the tongue; continuous tingling sensation; and allodynia, which is pain caused by a stimulus that does not normally provoke pain, for example, hot food or ice. However, diagnosing LN neuropathy can be challenging, with difficulty in determining if the disturbance is transient or permanent. Further, it is difficult to determine the need for surgical management and the postoperative prognosis. There remains a controversy regarding the appropriate time for surgical management in LN neuropathy, and no consensus has been established yet [7–9]. It is therefore essential to develop a diagnostic technique that can help identify the need for surgical management in the early stages of the nerve injury. Since 2000, we have diagnosed and managed more than 160 cases of LN neuropathy of varying severity developing after extraction of mandibular third molar. We have previously reported an outline of our management approach [10, 11]. The findings of our studies revealed that in case of transient paralysis, clinical neurosensory testing (CNT) results deteriorate immediately after tooth extraction and gradually recover, while Tinel’s test shows a negative result. Moreover, modern studies have proved that the persistent positive Tinel’s test may represent a poor sign of chronic peripheral neuropathy with neuroma formation. We propose a new method to differentiated between lesions with the ability to heal spontaneously and those that cannot be cured without surgery.

## Methods

We aimed to evaluate the utility of combining Tinel’s test and CNT in the early stages of LN injury to predict whether the lesion would heal spontaneously or require surgical management.

### Participants

This study was performed in accordance with the Declaration of Helsinki and was approved by the Wakayama Medical University Institutional Review Board (approval number 1699). Observational data were collected from patients who visited the Wakayama Medical University Hospital with unilateral LN injury after third molar extraction between May 2014 and March 2020. The included patients had no remarkable medical history, except for the LN disorder. Written informed consent was obtained from all patients before they underwent examination. For each patient, CNT and Tinel’s test were performed at every consultation. Patients were diagnosed with severe LN disturbance based on the findings of at least two detailed examinations performed at intervals. The interval between the first examination and the second examination was at least 1 month. We advised to add more examinations after the second examination if it's convenient. It is difficult to specify the indications for surgical management, but surgical management was performed if any of the following two conditions were met:

1. If the follow-up period after tooth extraction was less than 6 months, and no improvement, worsening of the condition, or allodynia were observed.
2. If 1 year had passed since the tooth extraction and the patient condition was poor or if the patient had allodynia.

### Diagnostic methods and criteria

**Fig. 1:**
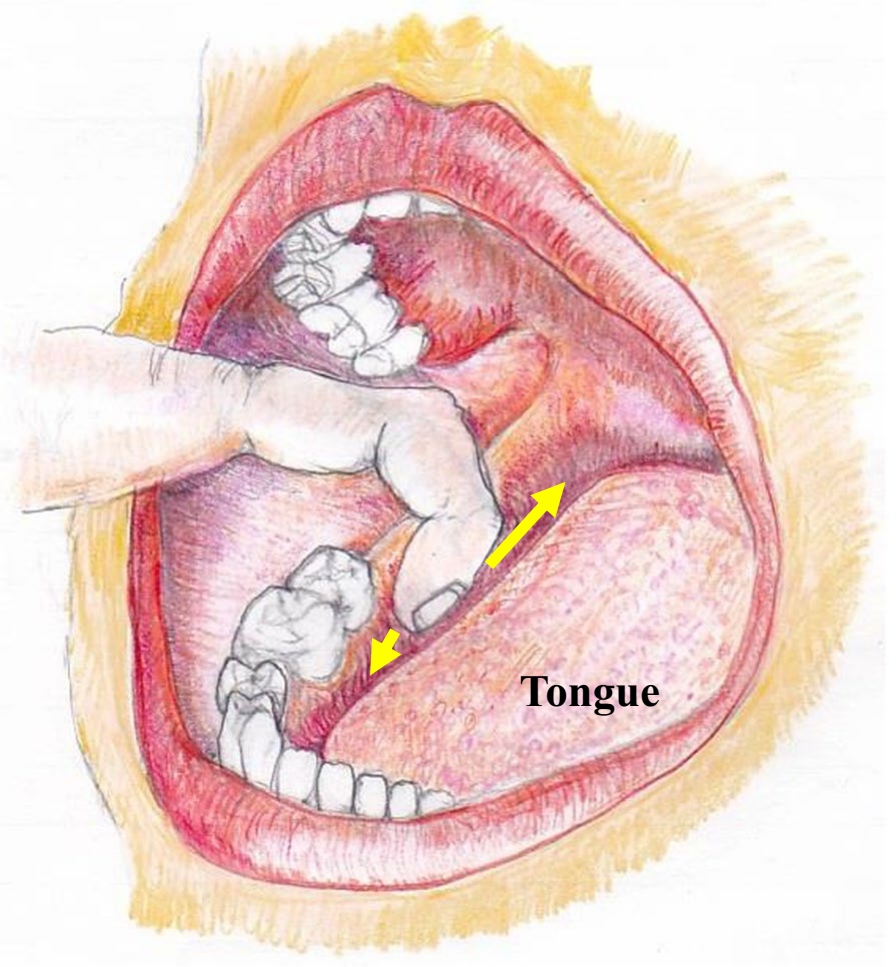
Manipulation of Tinel test

**Fig. 2:**
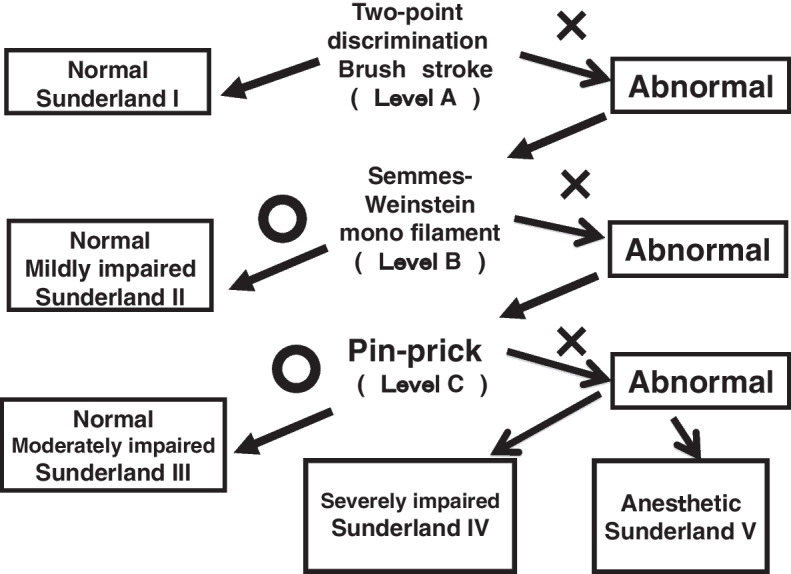
Flow diagram of Clinical neurosensory testing

Tinel’s test involved palpation of the lingual gingiva at the extraction wound site. The earliest timing to apply the Tinel’s test is when the extracted wound has been cured after sutures removal. It is important to palpate the lesion around the lingual gingiva adjacent to the mandibular third molar extraction socket with fingertips gently and without strong pressure in all directions as indicated by the arrows in Fig. 1. In the case of positive reaction, a tingling pain can often be observed running from the tongue tip to the tongue margin, the oral mucosa and the lingual gingiva from the molar to the premolar in the affected side. In rare cases, similar pain may occur in the affected lower submandibular lesion around submandibular gland. As a control, we should similarly palpate the lingual gingiva of the unaffected contralateral mandibular third molar to remind the patient that no pain occurs on the unaffected side. This maneuver helps to elicit a distal referred tingling or hyperpathia on the side of the tongue in the region of the injury (scores: 0, no sensation was recognized; 1, some pain recognized at the target tongue region). The other hand, CNT involves a three-stage inspection method as shown in Fig. 2. The second and third levels of the CNT test are performed depending on the response to the previous level. Flow chart of CNT presented in this study suggests the rational approach of first investigating the response of the thicker nerves and later examining the response of the thinner nerves. Practically, it is often observed that the functional recovery of the nerve fibers is initiated at the thicker nerve fibers and gradually progresses to the thinner fibers. Further, CNT can be easily performed at the bedside without requiring extensive equipment. Specific evaluation criteria for each level of CNT are described below.

#### Level A

Static two-point discrimination (2PD): before gentle contact with caliper tips is made on the lingual mucosae, the patient is asked to indicate when contact is felt and to identify whether that contact is of one or two points, which was then expressed in millimeters. Brush stroke directional sensation with a camel hair brush (brush) was examined by applying horizontal, vertical, and rotational stimulating movements on the lingual gingiva at the extraction wound site (scores: 0, no sensation was recognized; 1, sensations were recognized in only one direction; 2, sensations were recognized in two directions; 3, sensations were recognized during all movements).

#### Level B

Pressure pain threshold was examined using Semmens-Weinstein monofilaments (SWM) of 20 different diameters, with “1” referring to the smallest-diameter monofilament and “20” to the largest-diameter monofilament.

#### Level C

The pin prick test involved pricking the lingual gingiva at the wound site with a sharp needle (scores: 0, no sensation was recognized; 1, only pressure was recognized; 2, intensive pain was recognized).

### Statistical analyses

The Fisher’s exact probability test analysis was performed to identify correlations between changes in the Tinel test and CNT findings (2PD, brush, SWM, and pin prick). Two-sample Student’s *t* test was employed to determine the differences between the means of the variables measured within test groups. For all analyses, the statistical significance was set at *P* < 0.05. All data were statistically analyzed for significance using R version 4.2.1 (R foundation for Statistical Computing, Vienna, Austria).

## Results

### Participants

Thirty-three patients (29 women [88%], 4 men [12%]; mean age 35.5 years [range 16–67 years]) were included in this study. For all patients, the median interval between nerve injury and initial examination was 1.6 months (range 1–4 months). The median interval between nerve injury and the second examination for groups A (*n* = 10) and B (*n* = 23) before determining the need for surgical management was 4.5 months (range 2–20 months). There was no significant difference in the age distribution, sex, and timing of the first and second examinations after injury between the two groups (Table 1). The course of healing from the initial to final follow-up examination was monitored for every patient. These detailed findings are presented in Tables 2 and 3.

**Table 1:** Background of participants

	Group A	Group B	*p* value *1
Number	10	23	
Gender			
Male (%)	2 (20.0)	2 (8.7)	0.567
Female (%)	8 (80.0)	21 (91.3)	0.923
Age: year (SD)	35.20 (14.51)	35.74 (14.67)	
1st exam: month (SD)	1.20 ( 0.63)	1.78 (0.90)	0.074
2nd exam: month (SD)	4.20 (1.55)	4.74 (3.57)	0.652

*SD* standard deviation

The Fisher’s exact probability test analysis was used to search for correlations between gender

^*^^1^Student’s *t* test was employed to determine the differences between the means of the variables measured within test groups

**Table 2:** The patients who showed spontaneous healing tendencies (group A)

								Level A		Level A		Level B		Level C		
			1st exam	2nd exam	duration	Tinel’s test		2PD		Brush stroke		SW		Pin prick		Allodynia
No	Age	Gender	Months	Months	Months	1st Exam	2nd exam	1st exam	2nd exam	1st exam	2nd exam	1st exam	2nd exam	1st exam	2nd exam	
1	47	F	3	5	2	1	0	10 (5)	8 (5)	3	3	5 (1)	3 (1)	0	2	0
2	26	F	1	5	4	1	0	10 (5)	5 (5)	3	3	2 (1)	1 (1)	2	2	0
3	37	F	1	5	4	0	0	20 (5)	7 (5)	0	3	20 (1)	7 (1)	0	2	0
4	35	F	1	3	2	1	0	20 (5)	5 (5)	0	3	11 (1)	4 (1)	2	2	0
5	22	F	1	6	5	0	0	20 (5)	15 (5)	0	3	16 (1)	2 (1)	0	2	0
6	43	M	1	3	2	0	0	20 (5)	5 (5)	0	3	10 (1)	5 (1)	0	2	0
7	20	F	1	2	1	0	0	20 (5)	10 (5)	0	3	14 (1)	8 (1)	0	2	0
8	16	F	1	5	4	0	0	20 (5)	5 (5)	0	3	13 (1)	4 (1)	0	1	0
9	43	F	1	6	5	0	0	20 (5)	8 (5)	0	3	12 (1)	4 (1)	0	1	0
10	63	M	1	2	1	0	0	12 (5)	5 (5)	3	3	7 (1)	2 (1)	0	2	0

Gender: *F* means female and *M* means male. Tinel’s test: degree of pain perception (*0* means recognized not at all, *1* means recognized clearly)

*2PD* static 2-point discrimination. The values in parentheses are the opposite and control values measured by unit millimeter, *Brush stroke* brush stroke directional sensation; horizontal, vertical, and rotational stimulating movement were applied (*0* means recognized not at all, *1* means recognized only *1* direction, *2* means recognized *2* directions and *3* means recognized all movements). *SW* the Semmens-Weinstein monofilament is composed of 20 different diameter monofilament. 1 was assigned to the smallest-diameter and 20 was the largest-diameter monofilament. The values in parentheses are the opposite and control values

Pin prick: sharp touch with needle. (*0* means recognized not at all, *1* means recognized only slightly, *2* means recognize definitely) allodynia: *0* means recognized not at all, *1* means recognized clearly

**Table 3:** The patients who received surgical management (group B)

								Level A		Level A		Level B		Level C		
			1st exam	2nd exam	Duration	Tinel’s test		2PD		Brush stroke		SW		Pin prick		Allodynia
No	Age	Gender	Months	Months	Months	1st exam	2nd exam	1st exam	2nd exam	1st exam	2nd exam	1st exam	2nd Exam	1st exam	2nd exam	
1	67	M	2	6	4	1	1	20 (5)	20 (5)	0	0	15 (1)	15 (1)	0	0	0
2	22	M	1	4	3	1	1	20 (5)	20 (5)	0	0	15 (1)	15 (1)	0	0	0
3	32	F	1	4	3	1	1	20 (5)	20 (5)	0	0	12 (1)	16 (1)	0	0	0
4	40	F	2	4	2	1	1	20 (5)	20 (5)	0	0	15 (1)	13 (1)	0	0	0
5	18	F	2	4	2	1	1	20 (5)	20 (5)	0	0	12 (1)	13 (1)	0	0	0
6	41	F	2	3	1	1	1	20 (5)	20 (5)	0	0	12 (1)	14 (1)	0	0	0
7	52	F	2	8	6	1	1	20 (5)	20 (5)	0	0	16 (1)	20 (1)	0	0	0
8	25	F	1	4	3	1	1	20 (5)	20 (5)	0	0	12 (1)	11 (1)	0	1	1
9	37	F	1	4	3	1	1	20 (5)	20 (5)	0	0	8 (1)	12 (1)	0	0	0
10	36	F	1	3	2	1	1	20 (5)	20 (5)	0	0	12 (1)	12 (1)	0	1	1
11	33	F	2	4	2	1	1	20 (5)	20 (5)	0	0	9 (1)	18 (1)	0	0	0
12	50	F	4	6	4	1	1	12 (5)	15 (5)	0	0	8 (1)	9 (1)	0	0	0
13	25	F	1	20	19	1	1	20 (5)	20 (5)	0	0	12 (1)	15 (1)	0	0	0
14	51	F	2	4	2	1	1	15 (5)	18 (5)	2	2	8 (1)	11 (1)	0	0	1
15	18	F	3	5	2	1	1	8 (5)	18 (5)	0	0	8 (1)	11 (1)	1	1	1
16	36	F	1	2	1	1	1	20 (5)	20 (5)	0	0	15 (1)	19 (1)	1	1	0
17	25	F	1	3	2	1	1	20 (5)	20 (5)	0	0	16 (1)	19 (1)	0	0	0
18	60	F	3	4	1	1	1	20 (5)	20 (5)	0	0	13 (1)	15 (1)	1	1	1
19	59	F	3	4	1	1	1	18 (5)	18 (5)	0	0	7 (1)	10 (1)	0	0	1
20	25	F	3	4	1	1	1	15 (5)	20 (5)	0	0	10 (1)	11 (1)	1	1	1
21	18	F	1	2	1	1	1	20 (5)	20 (5)	0	0	13 (1)	13 (1)	0	0	0
22	32	F	1	4	3	1	1	20 (5)	20 (5)	0	0	15 (1)	15 (1)	0	0	1
23	20	F	1	3	2	1	1	20 (5)	20 (5)	0	0	12 (1)	15 (1)	0	0	1

Gender: *F* means female and *M* means male. Tinel’s test: degree of pain perception (*0* means recognized not at all, *1* means recognized clearly). *2PD* static 2-point discrimination. The values in parentheses are the opposite and control values measured by unit millimeter

*Brush stroke* brush stroke directional sensation; horizontal, vertical, and rotational stimulating movement were applied (*0* means recognized not at all, *1* means recognized only 1 direction, *2* means recognized 2 directions, and *3* means recognized all movements). *SW* the Semmens-Weinstein monofilament is composed of 20 different diameter monofilaments. 1 was assigned to the smallest-diameter and 20 was the largest-diameter monofilament. The values in parentheses are the opposite and control values

Pin prick: sharp touch with needle. (*0* means recognized not at all, *1* means recognized only slightly, *2* means recognize definitely) allodynia: *0* means recognized not at all, *1* means recognized clearly

### Tinel’s test and CNT

The patients were assigned to either group A or B. The spontaneous healing group (group A, *n* = 10) revealed a tendency for recovery within 6 months after tooth extraction. In seven cases, the Tinel test result was negative at the first inspection, and in three cases, the result changed to negative at the second inspection (Table 2). Further, although the CNT findings were initially deteriorated, they displayed a tendency for improvement later. None of the patients were diagnosed with allodynia. Conversely, the patients who received surgical management (group B, *n* = 23) underwent detailed examination at least twice within 20 months after the extraction but neither Tinel’s test nor CNT findings revealed a tendency for recovery. Further, the Tinel test result was positive for every patient in both examinations (Table 3).

### Correlation between the findings of Tinel’s test and CNT findings

Regarding the correlation between Tinel’s test and the Brush stroke and 2PD findings (CNT: level A), patients with a negative Tinel’s test in both examinations indicated a trend of improvement in CNT findings on subsequent inspection; conversely, patients with a positive Tinel’s test did not show a tendency for improvement in CNT findings. Regarding the correlation between Tinel’s test and SWM values (CNT: level B), the SWM value was normalized in cases of a negative Tinel’s test in the second examination, and conversely, patients with a positive Tinel’s test did not show a tendency for recovery. Regarding the correlation between Tinel’s test and the second pin prick reaction (CNT: level C), seven patients with a negative Tinel’s test at the first examination and three patients with a reaction in the second pin prick test with a negative Tinel’s test in the second examination showed a significant trend for improvement. Conversely, twenty-three patients with a positive Tinel’s test in both examinations could not reach score 2 (Table 4). In the second pin prick examination, eight patients perceived intensive pain and two experienced slight dull pain in the group that showed a negative Tinel’s test, whereas, in the group that showed a positive Tinel’s test, six patients experienced slight dull pain, and 17 displayed no reaction in the second pin prick test (Table 5). In the second pin prick test, in the group that showed a negative Tinel’s test, eight patients had a score of 2. Further, two patients perceived a score of 1, slight dull pain. In contrast, in the group that showed a positive Tinel’s test, 23 patients did not perceive intensive pain to pin prick test. Here, when the second Tinel’s test and CNT findings results up to level C were evaluated, patients showing a negative Tinel’s test and an improvement trend in CNT findings were consistent up to the level C stage. Considering the second pin prick reaction as the gold standard, we found that the sensitivity of Tinel’s test was 0.800, its specificity was 1.000, its positive predictive value was 0.800, and its negative predictive value was 1.000 (Table 6). As shown in Table 7, there was no correlation between the first Tinel’s test and the first CNT findings. As shown in Table 8, there was a correlation between the second Tinel’s test and second CNT findings. Furthermore, as shown in Table 9, the results of the first Tinel’s test and the second CNT findings were in good agreement, and the negative/positive results of the first Tinel’s test were reflected in the results of the second CNT findings (Table 9).

**Table 4:** Assessment of pin prick

	Variations of the 1st and 2nd Tinel's test			
	1st → 2^nd^	1st → 2^nd^	1st → 2nd	1
	Negative → negative	Positive → negative	Positive → positive	*p* value^*1^
2nd pin prick level 0 (%)	0 (0.0)	0 (0.0)	17 (73.9)	< 0.001
1 (%)	2 (28.6)	0 (0.0)	6 (26.1)	
2 (%)	5 (71.4)	3 (100.0)	0 (0.0)	

The Fisher exact probability test analysis was used to search for correlations between changes in the Tinel test

*^1^Student’s *t* test was employed to determine the differences between the means of the variables measured within test groups

**Table 5:** Assessment of 2nd pin prick in 2nd Tinel’s test

	2nd Tinel’s test		
	Negative	Positive	*p* value^*1^
2nd pin prick level 0 (%)	0 (0.0)	17 (73.9)	< 0.001
1 (%)	2(20.0)	6 (26.1)	
2 (%)	8(80.0)	0 (0.0)	

The Fisher exact probability test analysis was used to search for correlations between changes in Tinel’s test and pin prick

^*^^1^Student’s *t* test was employed to determine the differences between the means of the variables measured within test groups

**Table 6:** Diagnostic capability on 2nd Tinel’s test to the prognosis

Sensitivity	[95%, confidence]	0.800 [0.444, 0.975]
Sensitivity	[95%, confidence]	1.000 [0.518, 1.000]
Positive prediction value	[95%, confidence]	0.800 [0.444, 0.975]
Positive prediction value	[95%, confidence]	1.000 [0.789, 1.000]

**Table 7:** Correlation between Tinel's reaction and CNT. Correlation between 1st Tinel's test and 1st CNT

	Tinel's test				
	1st: positive *n* = 26		1st:negative *n* = 7		*p* value*1
1st 2PD (SD)	18.00		(3.78)	18.86 (3.02)	0.585
1st brush stroke (%) (grade 0 ~ 3, number)	0	23	(88.5)	6 (85.7)	0.635
	1	0	(0.0)	0 (0.0)	
	2	1	(3.8)	0 (0.0)	
	3	2	(7.7)	1 (14.3)	
1st SWM (SD)	11. 27		(3.56)	13.14 (4.18)	0.242
1st pin prick (%)(grade 0 ~ 2, number)	0	20	(76.9)	7 (100.0)	0.723
	1	4	(15.4)	0 (0.0)	
	2	2	(7.7)	0 (0.0)	

The Fisher exact probability test analysis was used to search for correlations between changes in the Tinelʻs test and each examinations

*SD* Standard deviation

^*1^Student’s *t* test was employed to determine the differences between the means of the variables measured within test groups

**Table 8:** Correlation between Tinel's reaction and CNT. Correlation between 2nd Tinel's test and 2nd CNT

	Tinel's test				
	2nd: positive *n* = 23		2nd: negative *n* = 10		*p* value*1
2nd 2PD (SD)	19.52		(1.20)	7.30 (3.23)	< 0.001
2nd brush stroke (%) (grade 0 ~ 3, number)	0	22	(95.7)	0 (0.0)	< 0.001
	1	0	(0.0)	0 (0.0)	
	2	1	(4.3)	0 (0.0)	
	3	0	(0.0)	10 (100.0)	
2nd SWM(SD)	14.00		(3.02)	4.00 (2.21)	< 0.001
2nd pin prick (%) (grade 0 ~ 2, number)	0	17	(73.9)	0 (0.0)	< 0.001
	1	6	(26.1)	2 (20.0)	
	2	0	(0.0)	8 (80.0)	

The Fisher exact probability test analysis was used to search for correlations between changes in the Tinelʻs test and each examinations

*SD* Standard deviation

^*1^Student’s *t* test was employed to determine the differences between the means of the variables measured within test groups

**Table 9:** Correlation between Tinel's reaction and CNT. Correlation between 1st Tinel's test and 2nd CNT

	Tinel's test				
	1st: positive *n* = 26		1st: negative *n* = 7		*p* value*1
2nd 2PD (SD)	17.96		(4.57)	7.86 (3.67)	< 0.001
2nd brush stroke (%) (grade 0 ~ 3, number)	0	22	(84.6)	0 (0.0)	< 0.001
	1	0	(0.0)	0 (0.0)	
	2	1	(3.8)	0 (0.0)	
	3	3	(11.5)	7 (100.0)	
2nd SWM (SD)	12.69		(4.67)	4. 57 ( 2.30)	< 0.0 0 1
2nd pin prick (%) (grade 0 ~ 2, number)	0	17	(6 5.4)	0 (0.0)	0.001
	1	6	(23.1)	2 (28.6)	
	2	3	(11.5)	5 (71.4)	

The Fisher exact probability test analysis was used to search for correlations between changes in the Tinelʻs test and each examinations

*SD* Standard deviation

^*1^Student’s *t* test was employed to determine the differences between the means of the variables measured within test groups

## Discussion

Maxillofacial surgeons sometimes encounter patients with trigeminal nerve sensory deficits after mandibular third molar extraction. Ghali and Epker suggested practical and objective approaches to assess these patients and diagnose nerve injury, potential for recovery, and need for secondary microneurosurgical intervention; they proposed the CNT as a diagnostic method in 1989 [12]. Zuniga et al. attempted to determine the statistical efficacy of CNT and identify a correlation between the sensory impairment score obtained by preoperative testing and the degree of nerve injury. The multisite, randomized, prospective, blinded, clinical trial was conducted among 130 patients with inferior alveolar nerve and LN injuries. A statistically significant positive relationship was found between the sensory impairment score and degree of nerve injury. Moreover, the efficiency of CNT was greater for diagnosing LN injuries rather than inferior alveolar nerve injuries [13]. However, the history of the Tinel test is even older. In 1915, Tinel, a famous orthopedic surgeon, first described this test for the extremities. He reported a tingling sensation or formication produced by slight percussion of a nerve trunk following an injury. The sensation radiates into the cutaneous distribution of the specific nerve and indicates the presence of regenerating nerve fibers [14]. Miloro described Tinel’s sign in LN disturbances in his textbook of Oral Maxillofacial Surgery as follows: palpation may induce Tinel’s sign, which is a provocative test of regenerating nerve sprouts that it is performed by light palpation over the area of suspected injury. This maneuver elicits a distal referred tingling sensation at the target site. This sign is thought to indicate small-diameter fiber recovery; however, it is poorly correlated with functional recovery and is often confused with neuroma formation [6]. Gregg described in his paper as follows: Hoffman and Tinel in 1915 described the clinical phenomenon of tingling and shock-like sensation that are elicited by digital tapping over distal portions of regenerating nerves. These clinical signs were felt to represent “the presence of young axons in the process of growing”. Subsequent experiences, however, have shown that the “Tinel’s sign” may be easily misinterpreted, and, rather than a positive sign of spontaneous neural regeneration, it may also represent a poor sign of chronic peripheral neuropathy with neuroma formation. Modern studies have verified that hyperpathic clinical signs can represent sites of sensitized neuroma-in-continuity, as well as chronically sensitized amputation-type neuromas [15]. Suhaym and Miloro reported that the odd improvement was 2.28 (95% confidence interval 1.05–4.98) in the 6-month breakpoint studies according to the meta-analysis analysis results of the early treatment timing of the LN [8]. Among the more than 160 cases of LN neuropathy that we have experienced in the past, in the case of negative Tinel’s test, majority of these cases indicated a negative reaction at the first examination. There were no cases in which the transfiguration from negative to positive Tinel’s test changed in more than 6 months later after LN injury as shown in Table 2. Therefore, 6 months after LN injury would be an appropriate period to pursue Tinel’s test response. Hillerup and Stoltze followed up 46 patients with LN injury who showed spontaneous healing, and found that the highest frequency of recovery was 6 months after LN injury. They concluded that patients should be monitored repeatedly for at least 3 months and not operated on until neurosensory function no longer improved [16]. They observed that patients with a positive Tinel’s test at the initial examination also indicated a positive reaction at the final examination and that their neurological dysfunction was severe. On the contrary, patients with a negative Tinel’s test displayed a good recovery tendency of the LN function. Our findings are consistent with those of Hillerup and Stoltz, and we identified some patients with remarkable spontaneous healing ability within 6 months after LN injury. Patients with a negative Tinel’s test at the initial examination showed a good tendency for improvement of their nerve function, which is in line with the findings of Hillerup and Stoltz.

In the study by Hillerup and Stoltz, the tendency of recovery was evaluated based on the total CNT score, including scores for the perception of tactile and thermal stimuli, localization of the stimulus, and 2PD. The relationship between negative Tinel’s test and changing CNT findings over time had not yet been investigated. Our findings confirm the meaningful tendency among patients with negative Tinel’s test toward resilience and recovery from neuropathy. This may be attributable to improvement in the thick sensory nerves assessed in level A followed by gradual restoration of the normal function of the finer sensory nerves. We carefully performed both Tinel’s test and CNT at least twice at different time points. A positive Tinel’s test and poor CNT findings indicates severe LN disorder. In contrast, a negative Tinel’s test and mild symptomatic improvement with each performance of CNT findings may indicate spontaneous healing of the injured LN. Therefore, this is a meaningful diagnostic tool for oral surgeons who experience the difficulty in determining the need and appropriate timing of surgical management. Both Tinel’s test and CNT can be easily performed together, and their routine use by oral and maxillofacial surgeons should be encouraged.

## Conclusion

CNT is an established and rational diagnostic criterion for determining the degree of trigeminal neuropathy. It may be supplemented with the Tinel test to identify LN injuries with the ability to heal spontaneously and distinguish them from those that require surgical management.

## Data Availability

The data is available from the corresponding author upon reasonable request.

## Abbreviations

LN: Lingual nerve
2PD: Two-point discrimination
SWM: Semmens Weinstein Monofilament
CNT: Clinical neurosensory testing
